# The interprofessional person, people and place framework for interprofessional education on the distributed training platform

**DOI:** 10.4102/phcfm.v18i1.5635

**Published:** 2026-10-27

**Authors:** Jana V. Müller, Elize Archer, Ian D. Couper

**Affiliations:** 1 Division of Rural Health (Ukwanda), Faculty of Medicine and Health Sciences, Stellenbosch University, Cape Town, South Africa; 2 Department of Health Professions Education, Faculty of Medicine and Health Sciences, Stellenbosch University, Cape Town, South Africa

**Keywords:** intersectionality, transformative learning, interprofessional education, distributed training, curriculum, interprofessional socialisation, rural training, health professions education

## Abstract

**Background:**

Distributed training in undergraduate health professions education offers challenges and opportunities. Benefits include integration into healthcare teams, continuity of care and meaningful contributions to local healthcare systems. However, challenges such as human resource constraints, limited clinical training expertise and social support issues persist. Interprofessional education (IPE) is often easier to implement in smaller rural clinical placements, although these challenges tend to be more pronounced in such contexts.

**Aim:**

This study aimed to explore IPE within rural training platforms and to develop an interprofessional person, people and place (IP3) framework to support collaborative practice.

**Setting:**

The study was conducted on two rural training platforms in South Africa involving undergraduate students from multiple health professions.

**Methods:**

A qualitative study with an interpretivist approach was used to explore IPE experiences. Sixteen semi-structured interviews were conducted with participants from five different health professions in 2022 and 2023. Data were analysed using thematic and narrative methods with support from an independent researcher to enhance reflexivity and rigour.

**Results:**

Students on rural multiprofessional training platforms experienced formal and informal interprofessional learning opportunities influenced by intersectionality and pedagogy that shaped their practice as graduates. Collaborative practice continued to be influenced by intersectionality in the workplace.

**Conclusion:**

The study resulted in the development of the IP3 framework, which emphasises contextual transformative learning and intersectionality across curricular, co-curricular and extracurricular levels.

**Contribution:**

This framework provides a foundation for institutions seeking to introduce or expand IPE during distributed clinical training. It also contributes to the theoretical understanding of intersectionality and transformative learning in IPE.

## Introduction

*The Lancet* Commission on health professions education (HPE) report published in 2010^1^ advocated for the training of collaborative, practice-ready health professionals through transformative learning to cultivate leadership, critical thinking and ethical reasoning. The Commission called for a systemic approach to educational reform, ensuring these principles were embedded at all levels of HPE.^1^ The importance of integrating interprofessional education (IPE) to prepare healthcare workers for effective teamwork and collaborative patient care was emphasised both in the original report and in a follow-up publication in 2022.^1,2^ The integration of IPE and transformative learning into HPE is seen as a way to develop adaptive professionals better equipped to address modern healthcare challenges and lead change in complex healthcare systems.^1,2^ The aim is for institutions of higher education to co-create a workforce that can lead the change required to build more effective, efficient and equitable healthcare systems.

With growing evidence of the positive effects of interprofessional collaborative practice (IPCP) on reducing medical errors, improving medication adherence and prescriptions, enhancing patient satisfaction and education, and optimising referral systems,^3^ embedding IPE into all HPE is aligned with best practice. Interprofessional teamwork has also been shown to reduce burnout and turnover of staff.^4^ Interprofessional collaborative practice is not the solution to all healthcare challenges, but it is clear from the literature that IPE has the potential to help build a workforce that can offer more efficient, effective, equitable and sustainable healthcare. For this reason, institutions of higher education should integrate IPE into curricula and practice in their efforts towards socially responsive HPE, where training meets the needs of the healthcare system.

In order to effectively equip healthcare professionals with the necessary skills and competencies not only to function within the current healthcare system but potentially to transform it, training institutions must understand the context in which their students will operate. Challenges in South Africa (SA), like many low- and middle-income countries, relate to an inequitable distribution of healthcare and social services, human resource constraints, the quadruple burden of disease, the high rates of medical error and litigation and high rates of burnout, to mention a few.^5,6,7^ Looking towards the future, the challenge for HPE lies in creating a responsive undergraduate IPE curriculum for a healthcare service (HCS) that is not yet prepared for the delivery of IPCP. This is especially pertinent, knowing that IPCP is not necessarily commonplace nor is it well understood in the workplace.^8^ Despite educators hoping that IPE will encourage graduates to introduce IPCP into the HCS, the question remains, how do educational institutions prepare students for IPCP in the South African healthcare context?

### Distributed training

The reconceptualisation of HPE in SA includes training students along the continuum of care in urban and rural communities.^9,10^ This type of training includes exposure to the broader HCSs outside of specialist hospitals and in patients’ local communities. In other words, this implies healthcare training that is distributed across levels of care, from here on referred to as distributed training. It is in these distributed training contexts that students are exposed to the patient and family, primary health care services, diverse communities and cultures, a variety of patient conditions and the experience of working together with several other professions.^11^

The documented benefits of remote distributed training include students feeling more integrated into the healthcare team, continuity of care with patients, access to other healthcare professionals on the platform^11,12^ and the demonstrated contribution final year students make to the local healthcare system.^13^ However, students training on remote platforms face challenges related to human resource constraints and the level of clinical training expertise available to them on the platform.^13^ Students also have problems related to social support when far removed from their existing support systems and networks that they may have established over time during their training at a central academic campus.^12^ The challenges of distributed training, across all levels of the HCS, are further compounded by the increasing complexity of patient diagnoses and the influence of the social determinants of health.^7^ Implementation of IPCP, in a healthcare system with the aforementioned complexities, is a valuable strategy to improve patient outcomes and provide the highest quality of care.^14,15^

### Interprofessional education

Interprofessional education, which aims to cultivate IPCP, occurs when students from two or more professions learn with, from and about one another to improve collaboration and the quality of care and services.^16^ Interprofessional education has been shown to be most effective when implemented at a practical level and it is often easier to implement in rural training environments where students from more than one degree programme are placed together in smaller HCSs.^17^ Unfortunately, IPE to facilitate IPCP varies across institutions, and evidence to support its value is predominantly from the Global North.^14^ Concerns that IPE is an add on and additional outcome to fit into an already full curriculum have been voiced,^18^ but the international and local calls to incorporate IPE into undergraduate and postgraduate training is clear and cannot be ignored. Understanding how IPE is fostered during remote distributed training and if it results in IPCP is crucial to conceptualise how IPE can be introduced at other distributed training sites or incorporated into existing learning opportunities.

This article, which presents a framework for IPE on distributed training platforms (DTPs) stems from work done for Jana V. Müller’s Doctor of Philosophy (PhD) research. The context of the PhD study is distributed rural clinical training platforms in SA that required students from different professions to live and work away from the main campus situated in the metro for periods of 8 weeks or longer. These students shared residences and worked across various sites, but in the same town. This context provided continuity with the community, which was fostered by immersive, distributed training in and with communities that is considered longitudinal.^19^

The PhD study used triangulation of different theories, namely, transformative learning, socio-cultural theory of learning and intersectionality, which provided a triple lens through which to understand how IPE during undergraduate education influenced IPCP as students and when they were first-year graduates. The data were assimilated into a proposed framework to assist with the incorporation of IPE into existing distributed training, and is presented as the interprofessional person, people and place framework (IP3 Framework). In this article, we will expand on this framework.

## Research methods and design

The overarching aim of the PhD research study was to explore the perceptions of medical and health sciences students undergoing rural, longitudinal multiprofessional placement regarding their experiences of IPCP as students and as graduates.

An interpretivist approach was used to conduct a qualitative research study that consisted of two phases. The first phase explored the interprofessional learning (IPL) experiences of 16 undergraduate students training on two rural multiprofessional training platforms in SA during 2022. The second phase explored these same participants’ perceived capacity to engage in IPCP as graduates at the end of their first year of work in the South African public healthcare sector during 2023.

### Data collection

A total of 32 semi-structured individual face-to-face interviews (except one interview in 2022 because of illness) were conducted by Jana V. Müller, and interviews took between 50 min and 90 min.

The students represented the following professional disciplines: Human Nutrition, Medicine, Occupational Therapy, Physiotherapy and Speech-Language and Hearing Therapy. These were the disciplines training at the two rural sites.

### Data analysis and reflexivity

Recorded data from the interviews were transcribed, anonymised and initially analysed inductively using ATLASTi^20^ by Jana V. Müller. During the research process, both thematic and narrative analysis of the data was conducted. Reflexivity played a large role during the data analysis because of the nature of the findings and the research approach adopted. Jana V. Müller and her supervisors, Ian D. Couper and Elize Archer, engaged in critical reflection on a regular basis. An independent researcher from a different professional and demographic background assisted with anonymised data analysis and reflexivity during both phases of the research study. Two academic colleagues from different professional and demographic backgrounds assisted with critical reflection during the write-up stage of the research.

### Ethical considerations

Ethical clearance to conduct the study was granted by the Human Research Ethics Committee (HREC) of Stellenbosch University (S22/04/069 [PhD]) and the relevant academic administrations. All participants provided written informed consent before both the 2022 and 2023 interviews, after the specifics of the research were explained in detail and their rights to withdraw without any consequence were explained.

## Results

The findings from the overarching study, presented and previously published across four papers, are summarised below. These relate to the intrapersonal, interpersonal and environmental factors influencing IPE and IPCP on the rural multiprofessional training platform.

Specific opportunities and experiences that promoted IPL during undergraduate training on a rural multiprofessional training platform were identified and have been described in detail.^21^ The IPL opportunities that were identified existed at curricular, co-curricular and extracurricular levels and depended heavily on the opportunity for interprofessional socialisation on the rural multiprofessional training platform. It was discovered that students’ own intersecting social identities influenced their interprofessional socialisation and experiences of IPL opportunities during their training.^22^ Students were deeply affected by situations that challenged or promoted their belonging based on a variety of social identities and not just their professional identity. Social identities related to language, ethnicity, religion and profession all played a role in student’s experiences of IPL and IPCP. This emphasised the need to consider the individual and interpersonal factors when exploring the data and that the recognition of inter- and intrapersonal factors that influence students’ engagement during explicit or implicit IPL is important.

Transformative learning theory was proposed as a possible pedagogy to facilitate students in the processing of their disorienting interprofessional experiences and understanding of the world and how they fit into it. Transformative learning requires critical reflection on disorienting experiences, which should be facilitated. This is especially true when students are confronted with the limitation of their worldview and they should be encouraged to consider and adopt new roles within their broader understanding of the world if necessary.^23^

However, the data demonstrated that not all students were able to reflect on and make sense of their experiences and therefore an understanding of the potential challenges educators and students may face when using transformative learning theory is recommended. This highlights the need for an ethical approach to facilitate transformative learning during distributed training which has been detailed in a separate publication.^24^

During the second phase of the research, when participants were followed up as graduates at the end of their first year of work, it was evident that they entered the workplace at the bottom of the existing hierarchy.^25^ This complicated their efforts to influence an HCS that had not necessarily adopted an interprofessional approach to patient care. Further barriers such as workplace culture and the absence of existing interprofessional initiatives affected graduates’ experiences of IPCP. However, pre-established relationships from their time on the rural multiprofessional training platforms and the ethos of holistic care fostered during training using the International Classification of Functioning, Disability and Health (ICF) positively influenced their engagement in IPCP. In some cases, graduates were able to initiate IPCP despite the challenges they faced. The findings from the research can be conceptualised in relation to the influence of the individual person, the people and the place on participants’ IPL and IPCP.

### Person

When discussing intrapersonal factors, we are referring to the individual; simply put, *the person* and how each individual’s intersecting identities influence their own IPE and transformative learning process. Personal factors that influenced IPCP identified through this research included: students’ desire to provide holistic patient care to their patients; their implicit bias of others; the ability to critically reflect or recognise their emotions when faced with a disorienting dilemma; or the influence of their social identities, that is, ethnicity or religion, on their IPL experiences.

The data demonstrated that participants and their intersecting social identities cannot be separated from their engagement with other people or the context in which they learn. Hierarchies that exist on the training platform are not only professional but also cultural, relating to language, ethnicity and religion.

### People

The notion of interpersonal refers to the engagement between individuals in social, academic and clinical spaces. Interpersonal refers to *the people* on the DTP and how their interactions influence IPE and IPCP. Factors of influence identified from this study were: the opportunity for students and graduates to interact socially across professions and build personal and subsequently professional relationships; belonging to a community of practice; the challenge of communication barriers and marginalisation by others.

The data demonstrates that pre-existing relationships not only encourage interprofessional engagement and collaboration in undergraduate training, but this collaboration overflows into the graduate space. This was especially true for graduates who were working in under-resourced settings, in environments where IPCP was not the norm, and where graduates faced challenges initiating IPCP.

### Place

The context refers to *the place*; this encompasses not only the physical training and living environment in which students are placed, but also the social and organisational culture that exists in that environment. The presence of structural hierarchy; the value of shared accommodation and transport in initiating interprofessional relationships and students’ exposure to structured practical IPE during training were all identified as influencing factors to IPCP.

Marginalisation and structural inequities existed during interpersonal engagement with an interprofessional team, but they were also perpetuated by the culture of the place in which students were training. An example of this was the language used during training by teaching clinicians that marginalised students who did not understand that language, as a result the student missed out on IPE.

The findings demonstrated that an IPCP culture can be achieved by focusing training on patient-centred care. The determination students had in wanting to make a difference in patient care acted as a motivator to formulate a holistic picture of the patients’ cases. Students sharing clinical training sites and patient cases encouraged interprofessional engagement.

### The intersection of person, people and place

Figure 1 provides a visual representation of the complexity of the overlapping and interacting nature of person, people and place during learning on the DTP.^26^ The Venn diagram in the centre of Figure 1, in hues of orange, represents the intersecting social identities of the student, which is overlapped by continuous and multiple engagements with the people on the training platform. These interactions with self and others are situated in the place where students are training, which has its own structural and cultural influences (represented by the outside circle). This figurative representation demonstrates that person, people and place cannot be separated from one another when considering IPE and should not be considered in isolation from one another when designing IPE opportunities.

**FIGURE 1:**
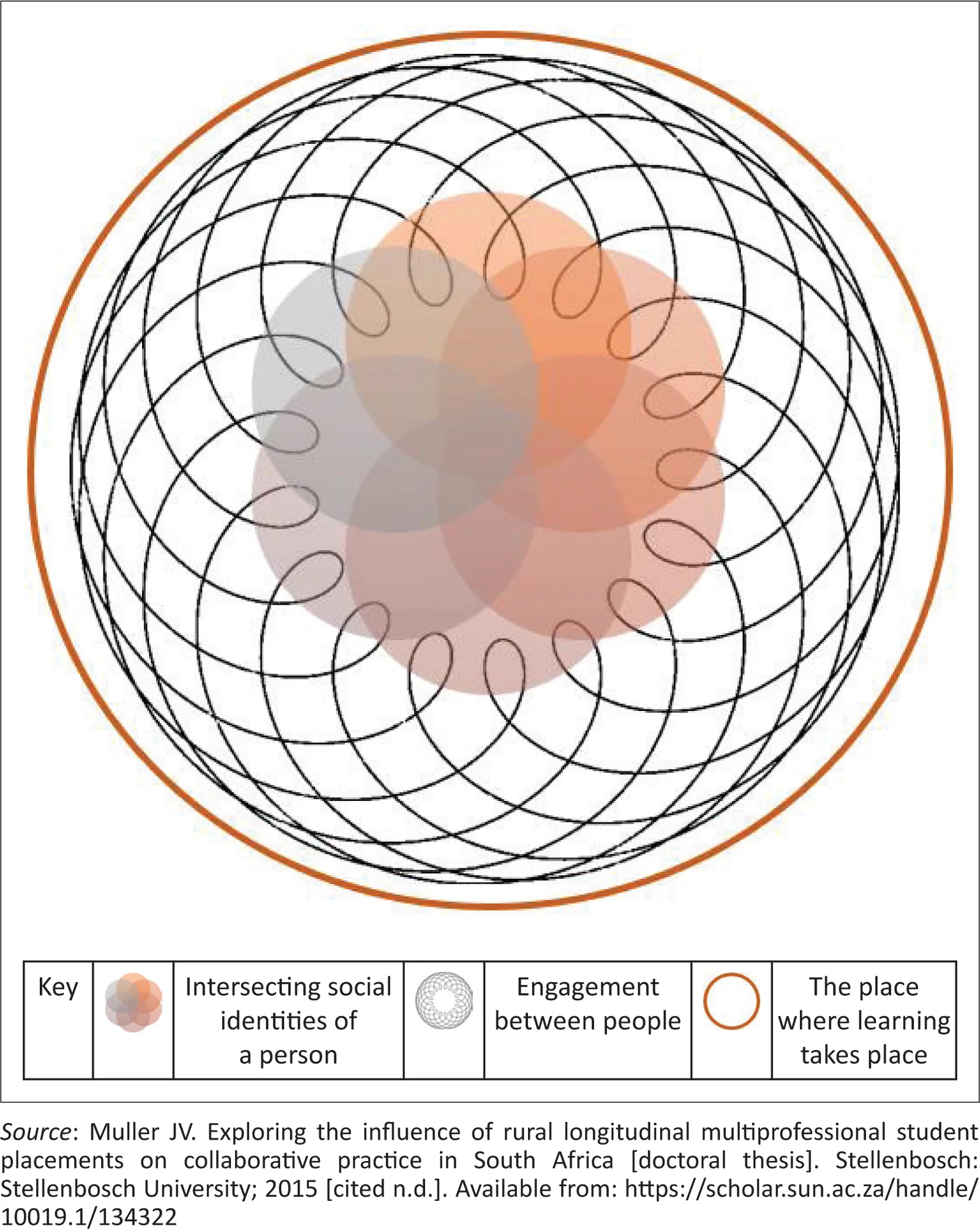
The overlapping and interconnected levels of influence on undergraduate students’ experiences of interprofessional education while training on the distributed training platform.^26^

The following section explores, in detail, how these findings can foster IPL on the DTP when considering them in light of person, people and place.

## Discussion

The findings of this research, summarised above, relate to the intrapersonal, interpersonal and environmental factors influencing IPE and IPCP. Based on these findings, strategies are required to best prepare our students, as well as the environment and structure in which they work, for the complexities of working interprofessionally.

The recognition of the importance of the person, their participation with others in the environment and the place where they train is not new in HPE.^27,28^ The interplay between the material structure of the system, the norms, values and workplace culture, and the agency of the student learning in that space is critical to what students gain from that learning experience.^29^ The findings of this study come to the same conclusion: the person, the people and the place are all of utmost importance when considering IPE. Building on this, we discuss possible strategies to overcome or capitalise on these factors to develop an IPE approach in the form of an IP3 framework, summarised in Table 1, that is responsive to the needs of the healthcare system. The framework is designed to guide the optimisation of existing or planning of new IPE experiences during training on the DTP.

**TABLE 1:** The interprofessional person, people and place framework: Summary of research findings and strategies to embed interprofessional education into remote distributed training.

Considerations	Challenges	Strategies
**Person**		
Intersectionality	Exclusion from learning opportunities based on social identities. Individuals are influenced by their intersecting social identities such as age, language, religion and culture which play a role in how they engage in their contexts and how their context engages with them.	Create opportunity for self-reflection of both students and staff to be critically conscious of positionality, power and privilege and identify their personal *loci* of responsibility. Facilitate the development of students’ (and staff) collective *loci* of responsibility when it comes to addressing issues of power and marginalisation in the training environment. Use critical reflection of case studies or stories to facilitate students’ and staff recognition of injustices and for them to develop a desire to make a difference.
Transformative learning	Disorienting dilemmas that arise related to students’ intersecting social identities. The potential for a halted transformative learning process or transformative trauma.	Empower students through the knowledgee of pedagogy: Be explicit about the following: ■ IPE is uncomfortable ■ The transformative learning pedagogy during training and the value of the disorienting dilemma ■ The challenges of the HCS and being placed in rural or remote areas for extended periods of time and how this may affect their support systems ■ The importance of recognising emotional responses in themselves ■ The importance of processing individuation during HPE (the emotional response students face when unconscious issues from their own experiences are triggered during training). Equip students with the necessary skills to put in place what they need to cope with some of these challenges. For example, teach emotional recognition and empathy through mentalisation.
**People**		
The need for relationship development	Lack of early exposure to other professions. Challenges in initiating IPCP in under-resourced settings.	Encourage earlier exposure to students from other professions in curricular, co-curricular and extra-curricular environments, so relationships can be built. Facilitate formal and informal interprofessional learning opportunities.
Power dynamics and marginalisation	Professional hierarchy is not the only hierarchy; age, language, religion and culture also play roles.	Use critical reflection of case studies or stories to facilitate students’ and staff recognition of injustices and develop a desire to make a difference in group work or workshops settings Encourage socialisation in terms of understanding culture, hierarchy through workshops, group work. Use the brave spaces framework and concept of difficult dialogues to encourage empathy and understanding.
The need for brave spaces	Psychological safety may not always be achievable. Differences in language, culture and gender.	Facilitate brave spaces using the brave spaces framework. Acknowledge and legitimise students’ diverse backgrounds. Role model emotional regulation and critical reflection.
Facilitate the practical reality of IPCP	Educators or clinicians are not role modelling IPCP with its challenges.	Gather insights from new graduates and local communities to understand the healthcare system students need to be trained for. Get feedback from or engage in reverse mentorship with graduates to understand the challenges of the South African healthcare system and the influence these have on IPCP. Role model IPCP or facilitate critical reflection of failed IPCP.
Supervision and mentorship	Overworked clinicians with limited capacity for facilitation. Lack of role modelling or active IPCP in the workplace. Students’ learning is facilitated by professionals in their discipline.	Facilitate interprofessional brave spaces with a trained facilitator who can role model and take on the responsibility for difficult dialogues. Facilitators should model interprofessional communication and conflict resolution and engage in professional advocacy during normal work responsibilities. Mentorship of students can be done by peers, staff or other clinicians, not always the supervising clinician. Use remote engagement or mentorship if face-to-face mentorship is not possible. Create contextually relevant, practical scenario-based communication, conflict and collaboration experiences that are role modelled by seniors
**Place**		
Practical and relevant exposure to IPCP	Students are being exposed to IPE in ideal contexts and not necessarily practical real-life scenarios. Students are being taught IPE separate to clinical practice.	Get students to reflect on what they observe in the healthcare system throughout their training, from early on, in light of the value of IPCP and whether or not it is taking place. Ensure lecturers are trained to live-out the challenge of IPCP when supervising students in the clinical space. Purposively select training sites where IPCP is happening or where there is receptivity to introducing it through student training. Tailor teaching strategies to focus on facilitators and barriers to IPCP in specific clinical contexts.
Create a culture of IPCP	Suboptimal understanding and limited appreciation of IPCP’s value in the South African healthcare system.	Create a culture of holistic patient-centred care using the ICF framework during normal clinical work. Use the ICF for patient assessment and management where students have to report on patient cases. Encourage and plan for a landscape of practice model (many communities brought together by a common purpose) through interprofessional supervision, where supervision is not profession specific. In other words, encourage a disruption of uni-professional facilitation which requires more human resources. Promote IPCP on the DTP through faculty development.
Power dynamics, marginalisation and structural inequalities	Marginalisation and structural inequities in training contexts.	Introduce intersectionality framework in the student curriculum. Introduce intersectionality at a faculty level so educators are equipped to evaluate structural hierarchies. Expose students to real-life challenges of IPCP in the clinical context, which include issues of power. Recognise power, privilege and responsibility as an institution in the context where students are being trained. Recognise and address inequalities in training contexts. Understand and mitigate power dynamics in the workplace.
Logistics and intentional multiprofessional placement	Lack of early exposure to other professions. Challenges in initiating IPCP in under-resourced settings. Individual programme curricula are not aligned with one another. Students are placed at different sites and rotate at different times.	Learning Have an IPE coordinator to encourage shared site placements and shared patients. Introduce the interprofessional socialisation framework throughout the curriculum. Facilitate formal and informal interprofessional learning opportunities at curricular, co-curricular and extra-curricular level. Find overlap in students’ outcomes to align learning opportunities, for example, graduate attributes, ethics, advocacy, patient portfolios. Facilitate IPE through shared clinical learning spaces. Get students to rotate through the same wards and present cases together. Allocate patient cases or wards to an interprofessional group of students. Formalise IPE by adapting existing assessments to incorporate an interprofessional approach. Living Encourage earlier exposure to students from other professions so relationships can be built. Encourage shared interprofessional residences and transport on the DTP. Find suitable DTPs for multiprofessional placements. Intentionally place students from different professions in shared accommodation. Plan for students to share transport to, from and around the environment Place students at the same site for longitudinal placements (longer than 8 weeks). Ensure the availability of information technology for remote engagement or mentorship if face-to-face mentorship is not possible.

*Source:* Adapted from Muller JV. Exploring the influence of rural longitudinal multiprofessional student placements on collaborative practice in South Africa [doctoral thesis]. Stellenbosch: Stellenbosch University; 2015 [cited n.d.]. Available from: https://scholar.sun.ac.za/handle/10019.1/134322

DTP, distributed training platform; IPCP, interprofessional collaborative practice; IPE, interprofessional education; ICF, International Classification of Functioning, Disability and Health; HPE, health professions education; HCS, healthcare service.

### Interprofessional person, people and place framework

The proposed IP3 framework is based on four fundamental elements and provides practical strategies to implement these considerations while recognising some of the challenges educators might face in doing so.

The four fundamental elements of the IP3 framework:

- Incorporation of contextual IPE at curricular, extracurricular and co-curricular levels on DTPs where students live and work.
- Consideration of students’ intersecting social identities in the contexts where the university places them to learn and recognition of students’ experiences of marginalisation during IPE.
- Pedagogically aligned facilitation of transformative learning on the DTPs is required, and this must be done in a way that avoids transformative trauma.
- Adaptation and alignment of IPE at undergraduate level to prepare students for the challenges they will face as new graduates wanting to engage in IPCP in the workplace.

### The interprofessional person, people and place framework explained

#### Person

**Intersectionality:** Research has shown that students need to be facilitated to develop critical consciousness through self-reflection so that they can be aware of their positionality (their privilege and power) and embrace their personal *loci* of responsibility.^30^ This is especially true in the multi-layered complexity of IPCP in a country with a history of discrimination and marginalisation. It is also clear from the study that professional hierarchy can no longer be the only type of hierarchy considered when exploring IPE and IPCP in context. Challenges (and facilitators) of IPE and IPCP also relate to age, language, religion and culture.^21,25^ Acknowledging this, exposing students to these realities and reflecting on the consequences can go a long way in preparing students for the real world.

This awareness and reflective practice can be achieved through critical reflection on case studies, poems, previous students’ reflections or stories to facilitate students’ recognition of injustices and prompt them to make a difference.^31^ These reflections can be held prior to students’ placement in order to sensitise them to some of the challenges they will face during training and working in the South African healthcare context.

Potter et al.^32^ used intersectionality to increase students’ self-understanding and perception, encouraging reflection on who they are and the experiences that have shaped them.^33^ However, Rehman et al.,^34^ caution that paying explicit attention to the core tenets of intersectionality (reflexivity, transformational identity and the analysis of power) is also important for educators in medical education to ensure the structural hierarchy and power dynamics of training environments do not marginalise students, we explore this further under ‘Place’.

Using findings from this PhD research, practical strategies on promoting intersectionality during DTP to enhance IPE are summarised in Table 1.^26^

**Transformative learning and trauma:** The disorienting dilemmas students experienced during training relating to person, people and place were deeply personal and, in some cases, painfully excluded students from learning opportunities. This cannot be ignored. Transformative learning is a useful theory for processing the disorienting dilemmas that arise during training when applied correctly, but incorrect implementation and a glib use of Mezirow’s theory carry educational consequences, such as transformative trauma or a halted transformation, if not used ethically during learning.^35^ As much as we teach our students to adopt a biopsychosocial approach to patients, as educators, we ought to model the same approach to our students. The consideration of being student-centred in the same way we teach our students to be patient-centred has been previously explored and the need to actively listen to what students’ learning needs are, in order to meet these needs is essential.^36^ The authors also urge educators to reflect in and on action, during the process of learning facilitation, in the same way we teach our students to reflect, so that we to can be change-agents in our own environments. The introduction and proper use of transformative learning theory and the intersectionality framework in the undergraduate curriculum may help conscientise students and educators to their positionality and awareness of their or others’ privilege and power through critical reflection.

Making students explicitly aware of the potential value of disorienting dilemmas in the process of transformative learning is important to enable students to take responsibility for their own learning. But this requires students to be able, among other things, to recognise emotion, examine these feelings, plan a course of action and reintegrate their new perspective into their life.

Facilitating the development of emotional intelligence, the awareness and assimilation of one’s own and others’ emotions to reflect and regulate responses, has the potential to mitigate healthcare worker burnout as a result of chronic stress.^37^ The need for students to learn to mentalise their emotions and understand others requires facilitation and an explicit understanding of how this relates to the transformative learning process. The educators who facilitate transformative learning need to be able to ask difficult questions while holding a space of trust and dialogue where students can recognise and embrace their transformation.^38^

#### People

**Relationship development:** The findings from this research demonstrated the value of relationships developed during undergraduate training, and its potential effects in IPCP even as graduates. There is a clear need for early exposure to other professions to foster relationship development. This can be facilitated by the introduction of the interprofessional socialisation framework across the curriculum for all students.^39^ Such socialisation can be started as early as the first year of training and can then be embedded across the years of training to facilitate relationship development and an interprofessional identity that can continue into the DTP.^40^ We emphasise the importance of creating opportunity, through informal IPL among students from different programmes, to develop trusting relationships.

Interprofessional socialisation is possible during training on or off the DTP without major changes in existing learning opportunities when consideration is given to the curricular, co-curricular and extra-curricular possibilities that exist in the environment. Interprofessional socialisation offers a useful framework to construct or plan IPL experiences and strategies to consider before and during DTP and are presented in Table 1 – the IP3 framework.

**Power dynamics and marginalisation:** A number of studies have demonstrated or recommended ways in which intersectionality can be introduced as a way of evaluating structural hierarchies in institutions and also to bring awareness to the importance of individuals’ intersecting social identities when it comes to interacting with one another.^41,42^ Rehman et al.,^34^ suggest methods like evaluating power structures and ensuring educators who consciously or unconsciously marginalise students are held to account. They advocate for breaking down teaching hierarchies to foster safe environments for discussing social categories and valuing lived experiences.

Eckstrand et al.,^42^ recommend implementing six strategies to encourage intersectionality using a focused, integrated and intersectional approach to diversity in HPE. These strategies are to:

[*E*]mbrace personal and collective loci of responsibility; examine and rectify unbalanced power dynamics; celebrate visibility and intersectional innovation; engage all stakeholders in the process of change; select and analyse meaningful metrics; sustain the commitment to achieving health equity over time.^42^

Practical recommendations aimed at encouraging intersectionality at an individual, interpersonal as well as an institutional level are presented in Table 1, based on findings from this research.

**The opportunity for brave spaces:** Brave spaces, where students can reflect on and process the disorienting dilemmas of facing marginalisation, power differentials and conflict, as well as the realities of the healthcare system, can play an important role in critical reflection and the processing of disorienting dilemmas, or trauma.^43^ Lackie et al.,^44^ emphasise the need to develop a culture of safety in IPE by facilitating psychological safety; while we accept that is a vital aspiration, and something for which we strive, we believe that we cannot expect students or even professionals to feel fully safe in the complexity of collaboration. Interprofessional education and IPCP not only face the challenge of professional hierarchies, but inevitably also hierarchies related to language, culture, age and gender, among others, that are inextricably woven into the health professions. If we wait for psychological safety before we collaborate interprofessionally, we may never move into tackling the important shared tasks of improving patient care and optimising HCSs. Similar to other researchers, we appreciate the notion of ‘brave spaces’ where students, regardless of their differences and previous experiences, are able to be in a place where these differences are acknowledged and legitimised.^43,45^ The structure of brave spaces requires the creation by a facilitator of a neutral space for learning and reflecting where students from different backgrounds and experiences can feel included.^43^ The diversity and influence of students’ prior experiences and backgrounds should be acknowledged and legitimised in these spaces. For this, social differences such as culture, hierarchy and diversity need to be incorporated into undergraduate training as highlighted by Bogossian et al.,^46^ who consider it essential to consider socialisation issues such as language and culture when planning IPE curricula. The introduction of a brave space during training can facilitate students’ critical reflection on their collective *loci* of responsibility when it comes to learning in systems of power and can provide opportunities for facilitators to role model their emotional regulation and critical reflective practice.

The hope is that brave spaces on remote DTPs will enable students to share stories and realities, question their own understandings of the world and build relationships that will follow them into their future practice.

**Supervision and mentorship:** Brave spaces, where students critically reflect on and share experiences and insights, should be facilitated by someone who can role model emotional regulation and awareness as well as the ability to reflect critically. Graduates in this study explicitly mentioned the need for positive role modelling of interprofessional collaboration, which can be difficult for educators to do if they are not familiar with the real clinical context and complexities of IPCP in practice.

Students navigating the complexities of an inequitable HCS require guidance through their transformative learning journeys. However, it is important to recognise that many clinicians are already overburdened and may lack the capacity or insight to effectively facilitate this process. In many DTP contexts, educators are stationed too far from learning contexts and do not provide supervision in clinical contexts, and students are dependent on clinical facilitators to learn. Facilitators cannot be compelled into IPE and IPCP unless they understand the value thereof and are supported in their role as clinical facilitators who need to help students achieve their outcomes.

To address this, educators can identify and empower clinical staff on the DTP capable of taking on this responsibility during site visits or accreditations for student placements. Additionally, workshops aimed at understanding transformative learning, the value of IPE and IPCP and planning and identifying opportunities to integrate the brave spaces framework could be beneficial.^43^ This entire process will necessitate formalisation and training from the faculty’s side.

Educator support of students through mentorship roles can be made possible by remote engagement. This mentorship can assist students in navigating the social, cultural or professional challenges that they might face on the platform. It is recommended in the literature that remote mentorship is most effective if the mentor and mentee have previously met face-to-face, and this would need to be coordinated where possible.^47^ When dealing with students who come from historically privileged or marginalised backgrounds, there is a need to develop a mentorship dynamic that is relational to encourage explicit conversations about discrimination or marginalisation during training. Mentors who come from a position of privilege run the risk of being blind to the challenges and needs of a mentee facing discrimination and prejudice, as are fellow students who may also be blinded by their perspectives of privilege. Difficult dialogues have been proposed as a mechanism to help optimise mentoring relationships by revealing biases and cultivating a sense of openness to change and dismantling systems that encourage marginalisation of students.^48^ These difficult dialogues can be facilitated by setting up ground rules and using the brave spaces framework, but ultimately mentors need to start with themselves in the development of a critical consciousness and understanding of their own worldview,^49^ which should be facilitated by faculty development initiatives.^50^

**Facilitate the practical reality of interprofessional collaborative practice:** Students should not be trained to collaborate in, and for, the perfect context, because it does not exist. As Mohamed et al.^51^ have reported, there is a suboptimal understanding of what IPCP is, and there is limited appreciation of the value thereof in the South African healthcare system.^8^ Students and educators, who are often removed from the clinical reality and complexity of the healthcare system, need to see this reality and face the challenges that exist in these spaces if they are going to make the change to interprofessional patient care that we are hoping they will make.

Our job as educators is to ensure we prepare our students to work interprofessionally even if the environment into which we are sending them is not yet engaged in IPCP. This means we have a responsibility, as educators, to understand the complexities that clinicians face in IPCP in the healthcare system. This is necessary if we are to promote and role-model IPCP during training. Students need to know and understand the challenges related to IPCP before they are asked to be different, this means educators require the skill to demonstrate contextually relevant IPCP. The use of transformative learning theory can provide a useful framework to construct learning opportunities that elicit the kinds of disorientation that working in the HCS does (conflict, hierarchy, time-limitations) prior to sending students to the DTP.

#### Place

**Practical and relevant exposure to interprofessional collaborative practice:** Designing student learning opportunities in line with the reality of the HCS requires insight into the existing challenges the healthcare system faces. The findings of this research showed that educators did not role model real-life examples of IPCP or contextually relevant IPCP experiences, because they were no longer familiar with or engaged in clinical contexts. Gathering information on the reality of multiple HCSs can be facilitated by new graduates who have real-life experiences in the system and who can offer insights into adapting IPE, while they are still familiar with the curriculum. Lyle and Greenhill,^52^ note that the local community where students are training can also provide input into the IPE students are exposed to, which can help educators and subsequently students better understand the local context and needs of the relevant population.

**Create a culture of interprofessional collaborative practice:** Having a holistic picture of a patient’s condition and circumstances lends itself to the realisation that patient care is complex, and it requires more than one profession to optimally manage a patient. A holistic insight into the biopsychosocial aspects of a patient can be facilitated using the ICF.^53^

The ICF framework is most effective in promoting IPCP when used by an interprofessional group of students.^54^ Students learning about the ICF framework in isolation from other professions do not appear to understand the importance and value of other professions.^55^

The data from this research demonstrated that at a curricular level, activities and student assessment that drove IPE were related to the use of the ICF, which is in line with other contexts.^56,57^ Research has also demonstrated that introducing the ICF in the Rwandan healthcare setting promoted IPCP,^58^ which supports the suggested strategy in Table 1 to introduce the ICF into the clinical environment and student assessment rubrics in the workplace.

Establishing or building landscapes of practice in the clinical environment has been recommended by Stalmeijer and Varpio^59^ to promote IPE. The notion of landscapes of practice, originally coined by Wenger-Trayner et al.,^60^ is considered to be an interaction between different communities of practice resulting in an interconnection of knowledge and learning. This cross-community of practice interaction encourages an interprofessional teaching dynamic in the training environment. Suggestions to enhance this in the clinical teaching context are for different professions to supervise different programmes, allowing students and clinicians or educators from different professions a chance to interact.^59^ Finding overlap in student outcomes and providing opportunities for shared facilitation by supervisors may facilitate the IPE component of training, normalise an interprofessional working environment (encourage role-modelling) and also alleviate some of the teaching burden. Placing students from different professions in the same clinical spaces and formalising IPCP by including it in students’ patient presentations and assessments can foster students’ initiative to engage with other professions. The collaborative behaviour of students may in turn change the culture of the context in which the students are training to become more open to and interested in IPCP, as has been demonstrated by other research conducted in SA.^19,57,61^

**Power dynamics, marginalisation and structural inequalities in the contextual environment:** As mentioned previously, it is important to recognise and identify the inequalities that bias some students and advantage others in the training context. This is necessary to conscientise students and educators to their role in mitigating marginalisation. Although educators may not be able to change the institutional culture of a healthcare facility, they cannot turn a blind eye to the inequalities students may face. It is imperative to understand why power is perpetuated in these contexts and what role faculty can play in changing that culture. This requires an enabling faculty development process that encourages difficult dialogues, encounters with others’ realities and self-reflection, which is in line with the recent recommendations by Van Schalkwyk and Blitz,^50^ for faculty development aimed at producing critically conscious graduates. Being explicit about the power imbalances that exist in the workplace that students will face is an important step in preparing students for the reality of the healthcare system.

**Logistics and intentional multiprofessional placement:** This research demonstrated the value of living and working in close proximity to students from other professions. A concerted effort needs to be made to find DTPs that can accommodate students from different professions both residentially but also for training purposes. Students should share residential spaces and transport to encourage relationship development and IPS. Where possible, students should be placed at the same site for extended periods of time^11^ and preferably allow for overlap of rotations; for example, physiotherapy, medicine and speech-language and hearing therapy all rotate for the 8-week (or longer) period at the same time during the year. In addition, identifying training sites on the DTP where there is sufficient space for students to be placed at the same site on the same day to allow for IPE and facilitation by academics or clinicians from different professions is important to create the recommended landscapes of practice.

Identifying DTPs that have existing multiprofessional opportunities, be they health facility- or community-based care, where IPE can be facilitated, would be optimal for student placement. Bogossian et al.,^46^ report that multiprofessional learning environments are crucial to prepare students for their role in a multiprofessional workplace.

The findings from this research and the proposed IP3 framework demonstrate that introducing or enhancing IPE into students learning need not take curriculum reform but can be embedded into existing curricular activities. This requires a novel way of approaching existing learning opportunities or learning spaces to optimise the engagement and education of interprofessional groups of students. This paper presents a framework from which to explore the incorporation of IPE into existing or renewed curricula on the DTP.

### Recommendations for future research

Once the IP3 framework has been piloted at a new or existing DTP, an evaluation of its value and influence will need to be conducted to determine its viability, influence and sustainability. This can be done using Margaret Archer’s structure, culture and agency framework, which also explores the notion of an individual’s agency, the culture where students are training (their interpersonal relationships) and the structure the students are training in.^29^

Identifying the structures that facilitate or hinder IPE at training sites where the IP3 framework has been used to introduce IPE could offer insight into challenges other DTPs might face when developing IPE programmes. Exploring the agency of students, educators and other stakeholders such as clinicians in terms of their motivations and behaviours to engage in or promote IPE can help with the implementation of IPE on the DTP. Evaluating the culture of the environment and people involved, and how this may change based on the improved understanding of intersectionality, will help determine the potential value of institutions committing to using intersectionality when it comes to exploring student learning and cultural changes in contexts. The insights gained from the structure, culture and agency framework can help us understand what works, for whom and under what conditions to help refine IPE on the DTP.

## Conclusion

It is not sufficient to place students on a DTP without thought and preparation for the personal, interpersonal and contextual challenges they will face, which will inevitably affect their interprofessional engagement. Universities need to adopt a strategy to facilitate the best possible scenario for IPE while considering the reality that students not only hail from different professions, but also different genders, religions, ethnicities and language groups.

Universities should acknowledge the intricacies involved when students with multiple social identities (person) engage with other students (people) during IPE in contexts that have their own structural power dynamics (place). One of the challenges in terms of acknowledging the role of intersectionality in IPE is how we adapt our teaching and the learning contexts to accommodate all students. Interprofessional education needs to be designed and facilitated in a way that acknowledges and responds to the challenges that exist within the individual, the learning context and the healthcare system.

Reflecting on the suggestions and possibilities for the facilitation of IPE on the DTP, there are expanded roles for both on-site clinical educators and academic faculty that are presented in the interprofessional person, people and place (IP3) framework for IPE.
